# Evaluating scintillator performance in time-resolved hard X-ray studies at synchrotron light sources

**DOI:** 10.1107/S1600577516002770

**Published:** 2016-03-24

**Authors:** Michael E. Rutherford, David J. Chapman, Thomas G. White, Michael Drakopoulos, Alexander Rack, Daniel E. Eakins

**Affiliations:** a Institute of Shock Physics, Blackett Laboratory, Imperial College London, London, UK; b Diamond Light Source, I12 Joint Engineering, Environmental, Processing (JEEP) Beamline, Didcot, Oxfordshire, UK; c European Synchrotron Radiation Facility, Grenoble, France

**Keywords:** dynamic imaging, shock compression, time-resolved, X-ray, scintillator

## Abstract

The short pulse duration, small effective source size and high flux of synchrotron radiation is ideally suited for probing a wide range of transient deformation processes in materials under extreme conditions. In this paper, the challenges of high-resolution time-resolved indirect X-ray detection are reviewed in the context of dynamic synchrotron experiments. In particular, the discussion is targeted at two-dimensional integrating detector methods, such as those focused on dynamic radiography and diffraction experiments. The response of a scintillator to periodic synchrotron X-ray excitation is modelled and validated against experimental data collected at the Diamond Light Source (DLS) and European Synchrotron Radiation Facility (ESRF). An upper bound on the dynamic range accessible in a time-resolved experiment for a given bunch separation is calculated for a range of scintillators. New bunch structures are suggested for DLS and ESRF using the highest-performing commercially available crystal LYSO:Ce, allowing time-resolved experiments with an interframe time of 189 ns and a maximum dynamic range of 98 (6.6 bits).

## Abstract 2

Scintillator performance in time-resolved, hard, indirect detection X-ray studies on the sub-microsecond timescale at synchrotron light sources is reviewed, modelled and examined experimentally. LYSO:Ce is found to be the only commercially available crystal suitable for these experiments.

## 1. Introduction   

The brilliance of synchrotron radiation enables the study of phenomena across a range of spatial and temporal scales, from diffraction experiments able to probe the dynamics of atomic structure to radiography techniques capable of resolving deformation at the microscopic level. By combining new mesoscopic experimental measurements of material deformation with leading numerical models, better-performing next-generation materials may be designed from the ground up.

In recent years, a number of studies (*e.g.* Luo *et al.*, 2012 ▸; Hu *et al.*, 2013 ▸; Chen *et al.*, 2014 ▸; Eakins & Chapman, 2014 ▸; Rack *et al.*, 2014 ▸; Kantor *et al.*, 2014 ▸; Kareh *et al.*, 2014 ▸; Karagadde *et al.*, 2015 ▸; Jensen *et al.*, 2015 ▸; and references therein) have began to exploit synchrotron radiation to provide some of the first observations of important damage mechanisms at the mesoscale. Radiographic studies report observations of the early stages of buried pore collapse (Eakins & Chapman, 2014 ▸), crack pattern formation and instability growth (Luo *et al.*, 2012 ▸; Jensen *et al.*, 2015 ▸), while diffraction experiments have identified remarkably complex phase behaviour in bismuth (Hu *et al.*, 2013 ▸).^1^ However, several authors highlight the challenges associated with bringing dynamic experiments to synchrotron environments, noting trade-offs between signal-to-noise ratio (SNR), time resolution, interframe time and image ghosting (Luo *et al.*, 2012 ▸; Eakins & Chapman, 2014 ▸).

As the required temporal resolution of dynamic X-ray experiments approaches microseconds and below, increasingly severe demands are placed on detector technology, often resulting in X-ray detection, not the X-ray source, limiting the temporal and spatial resolution realisable in experiments. The challenges of dynamic time-resolved X-ray detection are exacerbated at higher X-ray energies (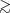
20 keV) where indirect detection *via* scintillating materials is in most cases a necessity (Gruner *et al.*, 2002 ▸). It is these higher energies, however, that permit the study of appreciable sample volumes (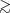 several mm^3^), which in turn allow longer-timescale processes, such as the nucleation and growth of new phases, to evolve and be stroboscopically examined. Accordingly, the choice of scintillator is crucially important to the temporal resolution accessible by experiment, and can be regarded as a significant bottleneck in the development of dynamic X-ray techniques.

In this paper, the challenges of time-resolved, hard, indirect X-ray detection on the sub-µs timescale are reviewed in the context of dynamic synchrotron experiments for the first time. A succinct review of scintillators suitable for X-ray detection in these experiments is then presented. As this discussion is tailored towards experiments recording sub-µs two-dimensional datasets (*e.g.* radiographs or diffraction patterns), it compliments recent reviews focused on less-rapid medical imaging scenarios (Nikl, 2006 ▸; Rack *et al.*, 2008 ▸; van Loef & Shah, 2014 ▸), and does not include photon-counting systems, which perform poorly in response to the large instantaneous fluxes expected in radiography experiments (Hatsui & Graafsma, 2015 ▸). Using the reported scintillation decay modes, the scintillator response at synchrotron light sources is modelled and validated against experimental data. The scintillator emission is then modelled for a range of bunch separations, allowing the usefulness of these scintillators to be evaluated for a number of existing bunch modes. New bunch modes are then proposed for dynamic experiments at Diamond Light Source (DLS) and European Synchrotron Radiation Facility (ESRF), optimized for existing detector and scintillator technologies. The X-ray energies of focus here (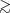
20 keV) make the conclusions of this paper most applicable to existing third-generation synchrotron light sources. However, it is intended that the discussions presented will be of use for detector development at the soon-to-be-online European XFEL (Roling *et al.*, 2014 ▸), LCLS II and MaRIE facilities (Barnes *et al.*, 2014 ▸).

Before continuing the discussion on indirect X-ray detectors, advances in time-resolved direct detector technology (Hatsui & Graafsma, 2015 ▸) should be noted. The development of detectors such as the Keck-PAD (Koerner & Gruner, 2011 ▸), which may resolve X-ray bunches of spacing on the order of ∼150 ns, and projects dedicated to resolving the 4.5 MHz pulse train at the European XFEL (LPD, DSSC, AGIPD) promise to see application in the experiments of interest here. However, their large pixel size (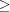
150 µm) and low efficiency for X-rays above ∼20 keV will deliver a limited spatial resolution and SNR, respectively, making these detectors less applicable to mesoscale problems such as spatially resolving grain-scale deformation or local phase transitions.

## 2. Time-resolved hard X-ray detection: challenges   

In this section, the challenges of time-resolved hard X-ray detection in dynamic synchrotron experiments are outlined and justified. Firstly, it is important to clarify the timescales of interest to a growing number of dynamic mechanical experiments at synchrotron light sources. Understanding the response of materials to the transient conditions experienced in the aerospace, advanced manufacturing, nuclear and defence industries, as well as astrophysical environments, requires subjecting samples to severe loading conditions, which may only be briefly supported. In these extreme experiments, samples are under loading for nanoseconds (laser-compression), hundreds of nanoseconds (pulsed power loading), <10 µs (gas-gun loading) and tens of microseconds (quasi-static loading) timescales. During such experiments, several important physical processes such as crack propagation and sub-surface defect generation evolve on timescales governed by material sound speeds, which are of the order of km s^−1^ (µm ns^−1^). For processes evolving at 1 km s^−1^ and an assumed system resolution of 50 µm (a value easily achievable with modern optically coupled systems), exposure times of no more than 50 ns are required if data are to be recorded without detectable motion blur. These short exposure times must be combined with interframe times appropriate to the loading conditions in order to reliably resolve material behaviour. For example, an interframe time of <1 µs is desirable in a gas-gun experiment lasting 5 µs.

Several factors contribute to the exposure time and temporal resolution achievable in dynamic synchrotron experiments:

(i) Synchrotron flux and bunch structure.

(ii) Detector sensitivity and gating capability.

(iii) Choice of scintillator material.

Across the many synchrotron facilities in operation there is a significant variation in storage ring energy, current and filling patterns, making some facilities far better suited to probing certain sample conditions over others. For example, the standard operating mode at the Advanced Photon Source (APS) (153 ns bunch separation) and the 4- and 16-bunch modes at ESRF (704 ns and 175 ns bunch separation, respectively), in combination with their high storage ring energies (7 GeV and 6 GeV, respectively), are already well suited to probing dynamic experiments with sub-µs temporal resolution. More continuous bunch structures can be modified to be better suited to dynamic experiments through bunch structure shuttering or chopping. A three-wheel chopper design has been demonstrated on ID09B, ESRF, which is able to isolate a single bunch from any of the ESRF bunch structures (Cammarata *et al.*, 2009 ▸), and, more recently, a chopper capable of delivering 50 ps, 8 keV pulses with a 1.25 MHz repetition rate has been developed at BESSY II (Förster *et al.*, 2015 ▸). More complex shuttering techniques, such as phonon Bragg switching (Bucksbaum & Merlin, 1999 ▸), continue to be developed as a method to isolate single X-ray bunches (Gaal *et al.*, 2014 ▸). Currently, the engineering and technical challenges, particularly the complications due to a high heat load, associated with bunch structure chopping means they are not yet commonplace on synchrotron beamlines, and thus have not been included in this review. In contrast with dedicated bunch modes or chopping, the BESSY-VSR project (HZB, 2015 ▸) intends to provide the option of a high average flux or 500 MHz pulse trains *via* new accelerator technology. Due to the amount of ongoing research in this area, a similar review of scintillator performance in the near future when these new techniques are more widely available would be of benefit to the dynamic loading community.

The sensitivity and exposure capabilities of several commercial cameras [*e.g.* Princeton Instruments PI-MAX4 and PCO Dicam Pro: ∼50% peak quantum efficiency (QE)^2^, ≥2.81 ns exposure time] have been demonstrated to provide sufficient SNR in dynamic synchrotron experiments (Wang *et al.*, 2008 ▸; Luo *et al.*, 2012 ▸; Eakins & Chapman, 2014 ▸). Thus, with a suitable bunch structure and detector, the choice of scintillator material is key to the temporal resolution and signal-to-noise levels accessible in a time-resolved experiment. In this paper, the discussion focuses on the influence of the scintillator and does not analyse the required SNR or dynamic range in an experiment, as the latter may vary significantly from high-resolution quantitative studies to more forgiving qualitative analyses.

## 3. Scintillator materials   

For data collection on the sub-µs timescale at synchrotron light sources, the scintillator material must meet the following four conditions:^3^

(1) *A high stopping power* to absorb an appreciable fraction of the incident X-rays. The experiments of interest here demand high spatial resolution (*e.g.* 1–100 µm) as well as high temporal resolution. The optical systems required to achieve this level of spatial resolution have depths of fields spanning micrometres to hundreds of micrometres. To retain this spatial resolution with the use of a scintillator the crystal must be no thicker than the system’s depth of field, assuming a single-crystal scintillator is used. These crystal thicknesses, however, come at the expense of X-ray absorption cross section, thus requiring a high stopping power to absorb and convert as many of the X-rays as possible. An attenuation length of <200 µm at 25 keV is taken as a lower bound in this review.

(2) *Large light yields* to provide acceptable signal-to-noise levels. The required short exposure times (*e.g.* 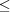
50 ns) have the potential to introduce significant photon counting noise, meaning a high conversion efficiency is required to maximize SNR. Based on recent leading dynamic synchrotron experiments which recorded data with a single bunch exposure (Luo *et al.*, 2012 ▸; Eakins & Chapman, 2014 ▸; Rack *et al.*, 2014 ▸), an efficiency of 20 photons per absorbed keV is taken as a lower bound on efficiency.

(3) *Rapid scintillation decay modes* to avoid the accumulation of afterglow due to periodic excitation. The time available to collect the scintillator emission in a single-bunch experiment is limited by the employed bunch structure. For example, a collection time of 704 ns is permissible at ESRF in the 4-bunch mode. Suitable scintillator materials must in general exhibit a primary decay mode with a characteristic time of <100 ns to avoid the build-up of deleterious background intensity between bunches. Otherwise, data will be compromised by motion blur and ghosting artefacts.

(4) *Visible, rather than ultraviolet, emission* for efficiency and flexibility in optical coupling. The QE of the required detectors drops off significantly for wavelengths <400 nm (∼20% at 350 nm for the Princeton Instruments PI-MAX and PCO Dicam Pro), which poses a large issue with respect to SNR. Furthermore, the transmission and imaging performance of optical relays, required to couple the scintillator emission to the detector, are, in general, severely reduced in the UV due to absorption and refractive index limitations, respectively. Therefore, visible scintillator emission ensures superior signal-to-noise levels and imaging performance.

Criteria (1), (2) and (3) combine to allow a sufficient SNR with a single-bunch X-ray exposure, which is typically of the order of 100 ps. This exposure time is, crucially, fast enough to permit high spatial resolution data collection without motion blur in extreme deformation scenarios such as imaging 10 km s^−1^ processes at 1 µm resolution.

Table 1 ▸ lists scintillator materials meeting the criteria above, and the parameters of relevance to time-resolved experiments. Of these materials, only three, YAG:Ce (Ce-doped Y_3_Al_5_O_12_), LuAG:Ce (Ce-doped Lu_3_Al_5_O_12_:Ce) and LYSO:Ce (Ce-doped Lu_2–*x*_Y_*x*_Si_2_O_5_:Ce), are available commercially, with the others still under development. LYSO:Ce is the best-performing readily available crystal with a high density and stopping power, large light yield and rapid (τ = 41 ns) single exponential decay term.

The most promising scintillator crystals under development are the metal-iodides (*e.g.* YI_3_, GdI_3_, LuI_3_). These crystals exhibit a good stopping power (attenuation length of <200 µm for 50 keV), green emission, high efficiency (90–115 photons keV^−1^) and fast decays (primary decay modes <50 ns). However, it is important to acknowledge the influence of the growth process and scintillator form (ceramic, single crystal, film, columnar) on the performance. Micro-columnar scintillators offer an improved effective efficiency as they channel the isotropically emitted light towards the scintillator rear surface, allowing the possibility of using a slightly thicker crystal while preserving spatial resolution. Single-crystal films allow access to doping concentrations not possible in the Bridgman and Czochralski methods and often display fewer long-lived decay components due to the absence of antisite defects (Martin *et al.*, 2009 ▸). However, the robustness of existing single-crystal production methods means that single crystals are still preferred for the hard X-ray studies of interest here where the required thicknesses on the order of 100 µm can be easily achieved *via* polishing.

## 4. Model   

The temporal evolution of the scintillator emission in response to repetitive X-ray excitation was modelled numerically. The train of X-rays emitted by a synchrotron was modelled in time according to the chosen synchrotron period and bunch mode. Every bunch was assigned to a time, 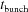, assumed to have unit intensity, and given a Gaussian temporal profile, which was chosen to approximate the measured temporal profiles (Wulff *et al.*, 1997 ▸, 2007 ▸). The emission of intensity from a given scintillator was modelled as a series of exponential decay processes. For a given scintillator, the number of exponential decay terms, the decay constants and their relative weighting were obtained from the literature; the constants used are summarized in Table 2 ▸. At a time *t*, the intensity emitted by the scintillator was calculated as the sum of intensity generated by all preceding bunches. Mathematically, this is given by equation (1):
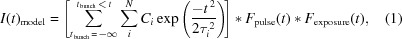
where the sum is over the *N* (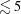) scintillation modes in a crystal, each of which having a weighting constant, 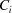 (<1), and decay constant, 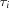 (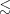
ms). The scintillator decay is convolved with 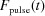, which accounts for the temporal shape of the synchrotron pulses, and 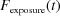, which accounts for the finite exposure time used to measure the decay response.

By summing the contributions of previous bunches in this way, the effect of repetitive X-ray excitation was captured; scintillation modes with decay constants significantly longer than the bunch separation have little chance to decay, creating an increasing background. In all cases, the model was run for a sufficiently long time (typically up to 100 synchrotron periods) so that the background reached a steady-state value and, thus, an equilibrium state to compare with the experimental data. Notably, 100 synchrotron periods is of the order of hundreds of microseconds to 1 ms, much less than the opening time of typical fast X-ray shutters (∼50–200 ms), meaning the influence of long-lived decay modes will be significant in shuttered synchrotron experiments. It was assumed that the referenced decay modes represent the scintillation process in the materials independent of supplier, and that the X-ray excitation process studied in this work did not change the overall scintillation modes.

## 5. Validation of the model   

### 5.1. Experimental method   

To validate the modelled scintillator response, a number of experiments were performed on beamline I12, DLS (Drakopoulos *et al.*, 2015 ▸), and beamline ID19, ESRF (Weitkamp *et al.*, 2015 ▸). At DLS, experiments were performed with the monochromatic beam (55 keV, 0.05% bandwidth). At the time of the experiments, the Diamond Light Source was operating with a reduced bunch current of 234 mA due to a preceding RF-cavity failure. A 686-fill bunch mode was used for the decay curve measurements. Per revolution, this bunch mode delivers a train of 686 adjacent bunches of 0.34 mA, separated by 2.0 ns. The remaining 250 buckets are unfilled meaning a 500 ns gap follows the pulse train. The scintillators and thicknesses examined on I12, DLS, are listed in Table 3 ▸.

The scintillator emission was relayed to a PI-MAX4:1024i ICCD camera (Princeton Instruments) *via* a pair of back-to-back achromatic doublets (AC508-075-A-ML, Thorlabs, 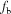 = 61.7 mm) operating at numerical aperture (NA) = 0.38. The camera was equipped with a Gen III filmless ‘HBf’ photocathode with QE of 35% at 400 nm, 50% at 500 nm and 35% at 700 nm.

The camera was synchronized with the DLS bunch structure *via* the RF bunch clock, in a similar manner to that described previously (Eakins & Chapman, 2014 ▸). Following receipt of the RF bunch clock as a trigger, a decay scan was performed by capturing a series of images (*e.g.* 1000) with a short (*e.g.* 5 ns) exposure time and an increasing RF-to-exposure delay. The intensifer gain was set to 100 in order to maximize signal during the short exposures.

A series of decay scans were also performed at ESRF. Scans were performed on beamline ID19, ESRF, using the 4-bunch mode (4BM) and 16-bunch mode (16BM). Per revolution, the 4BM delivers four 140 ps bunches of up to 10 mA each separated by 704 ns, and the 16BM delivers sixteen 140 ps bunches of up to 5.6 mA each separated by 175 ns. The scintillators and their thicknesses examined on ID19, ESRF, are listed in Table 4 ▸. The scintillator emission was relayed to the same PI-MAX camera, operating with a gain of 100, using back-to-back Hasselblad HC 2.2:100 mm lenses (NA = 0.15).

Decay scans were synchronized similarly to those at DLS. The ID19 beam (U17-6c undulator, filtering: 1.4 mm diamond, 2.8 mm aluminium) was used. The single-harmonic undulator, U17-6c, was used to minimize heat load delivered to the scintillators. Heat load was further controlled in the 16BM scans, which employed a 200 ms X-ray shutter. The spectral flux delivered by this beam configuration was dominated by a peak at 17.8 keV. All the decay scans in this work used a small beam size, ∼2.8 mm × 2.8 mm, to further reduce heat load to the scintillators.

## 6. Results   

**Figure 1:**
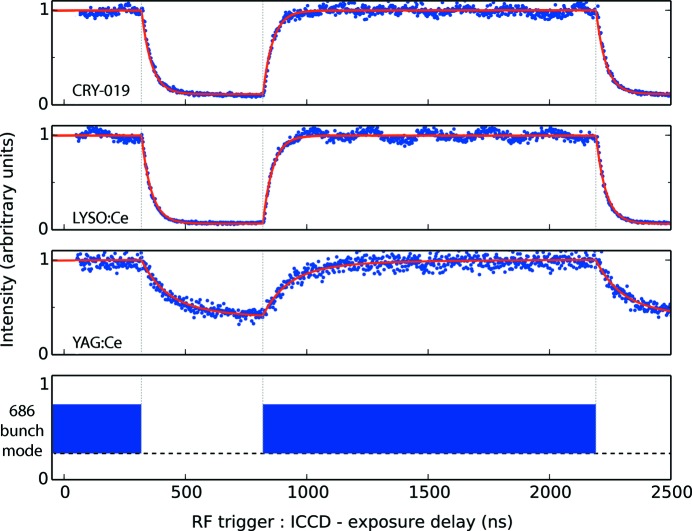
Experimental and fitted decay curves collected on beamline I12, DLS, with the 55 keV beam in the 686-bunch mode. Data for each scintillator are shown in a separate sub-plot. Experimental data are shown in blue. In each case, the intensity range of the curve has been normalized to its maximum, collapsing the values to the range of 0–1 to aid comparison between the different materials. The modelled data, using the constants in Table 2 ▸, are shown in red. The bottom sub-plot shows an illustration of the 686-bunch mode, indicating when the X-rays were incident on the crystal. Dashed grey lines mark the start and end of the 686-bunch train on the experimental and modelled curves. All three experimental scans comprised 1000 frames over 2550 ns with a 5 ns exposure time.

Figs. 1 ▸ and 2 ▸ show, respectively, decay curves collected for the studied scintillators on DLS and ESRF. Details of the individual scans (number of frames, averaging) are noted in the figures. In these figures the intensity values in each scan have been normalized to the maximum value in each scan so that the traces may be compared on the same scale. The performance of each scintillator is summarized by the measured dynamic range, defined as the ratio of the peak intensity to the minimum intensity in the measured response. The measured maxima, minima and dynamic range values (before normalization) are shown in Appendix *A* [App appa]. Because these dynamic range values are influenced by the experimental conditions (incident flux, choice of lens, *etc*.) they should not be used as an absolute guide of scintillator performance.^4^

Early work showed that on the timescales of interest here (tens to hundreds of nanoseconds) the pulse duration did not affect the modelled decay response. Furthermore, in some cases, the equilibrium background intensity produced by the model could not reproduce the background observed in experiment. Therefore, the modelled scintillator response was fitted to the experimental decay scan curves by varying *K* and *D* in equation (2):
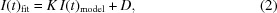
where *K* is a time-independent scaling factor accounting for the efficiency (*e.g.* incident flux, detector sensitivity) of the experimental system, and *D* is an additive constant matching the apparent background.

Overlaid on each curve in Figs. 1 ▸ and 2 ▸ is the scintillator response modelled using equation (2). The magnitude of the experimentally measured background and the inclusion of the constant *D* is discussed further in §7. The constants *K* and *D* were fitted in a least-squares manner with *C* and τ fixed to the literature values.

## 7. Discussion   

It was expected that the experimentally observed background offset was a result of the repetitive excitation of the scintillator on a timescale quicker than the dominant decay modes. Therefore, crystals such as LYSO:Ce should accrue almost no background between pulses whereas crystals such as LuAG:Ce should develop a large background offset, characterized by a lower dynamic range. Although this trend was observed, the modelled scintillator response did not reliably match the experimental background, indicating there are additional contributions to the observed background in experiment. Several experimental factors could have contributed to the anomalous background offset recorded in this work. Parasitic light signals or camera errors (thermal sensor drift, electronic offsets) could have contributed to the unexplained offset. Secondly, undesired electrons in adjacent bunches could have been a small source of persistent excitation, although with operating contrast levels of towards 10^−10^ this is unlikely to be a dominant factor (Rack *et al.*, 2014 ▸). Thirdly, thermoluminescence could have contributed to the measured discrepancy. To assess this further, the highest heat load scenario was considered. This was the experiment performed on LYSO:Ce in the 4-bunch mode at ESRF due to the large flux delivered by ID19 and the absence of an X-ray shutter during these scans. The temperature increase in LYSO:Ce during this scan, accounting for periodic X-ray heating and cooling *via* scintillator emission and blackbody emission, is calculated to be less than 10 K. At ∼310 K the thermoluminescence glow curves of LuAG:Ce (Nikl *et al.*, 2014 ▸), LYSO:Ce (Blahuta *et al.*, 2011 ▸) and YAG:Ce (Zych *et al.*, 2000 ▸) are non-zero suggesting that thermoluminesence could have been a small source of spurious background counts. However, the reduction of thermoluminescence due to dopant materials, which are understood to be included in the commercial crystals used here, is also reported (Blahuta *et al.*, 2013 ▸). Thus, quantifying the effect of thermoluminescence here is challenging. Finally, the significance of slowly decaying scintillation modes has been shown to increase with increased X-ray exposure time (Koch *et al.*, 1999 ▸; Martin *et al.*, 2009 ▸). As the exposure times in this study ranged from 200 ms at ESRF in the 16BM to minutes at ESRF in the 4BM and DLS in the 686BM, an increased weighting of long-lived components may have contributed to the background seen in experiment but not in the model. Because the relative contributions of these effects could not be retrieved from the experimental data collected to date, curves were fitted with a simple additive constant, rather than any additional time-dependent functions. As such, it is important to note that the modelled data represent a lower/upper bound on the expected background/dynamic range between bunches.

**Figure 3:**
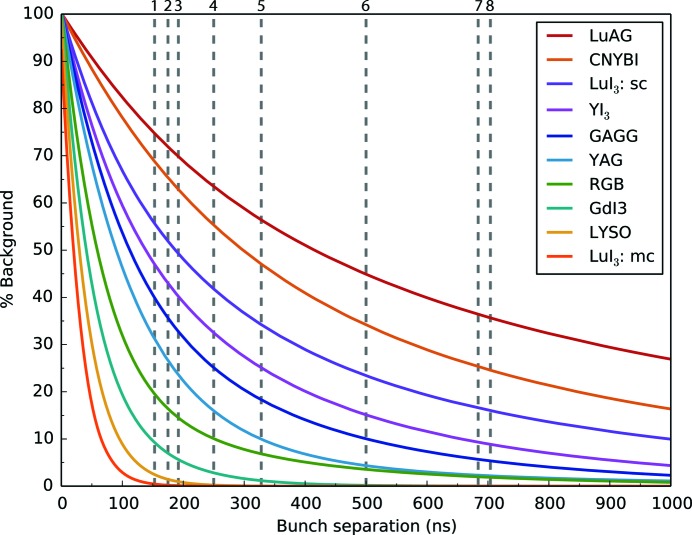
Graph of calculated background significance *versus* bunch separation. The graph shows the effect of bunch separation (from 100 ns to 1000 ns) on the usable portion of a dynamic signal, calculated from the modelled scintillator response. As the bunch separation is decreased (decreased possible interframe time) the scintillator emission has less time to decay, leading to a larger accumulated background between bunches and poorer contrast. LuI_3_:sc and LuI_3_:mc refer to the single-crystal and micro-columnar forms of LuI_3_, respectively. The dashed grey lines show a representative set of bunch separations in use at synchrotrons for time-resolved studies. From left to right these are: (1) APS, standard mode: 153 ns; (2) ESRF, 16-bunch mode: 175 ns; (3) PETRA III, time-resolved mode: 192 ns; (4) DLS, hybrid mode: 250 ns; (5) ALS, 2-bunch mode, 328 ns; (6) DLS, 686 mode: 500 ns; (7) SPring-8, 1/7 + 5-bunch mode: 684 ns; (8) ESRF, 4-bunch mode: 704 ns.

**Figure 4:**
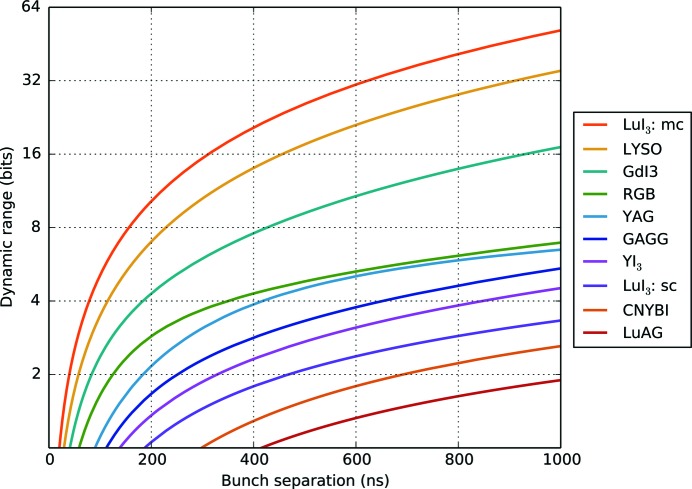
Graph of calculated available dynamic range *versus* bunch separation. Dynamic range is displayed on a log base-2 scale for comparison with standard CCD bit depths. The graph shows the effect of bunch separation (from 100 ns to 1000 ns) on the maximum dynamic range available in an experiment, calculated from the modelled scintillator response. As the bunch separation is decreased (decreased possible interframe time) the scintillator emission has less time to decay, leading to a reduced dynamic range between bunches and poorer contrast. LuI_3_:sc and LuI_3_:mc refer to the single-crystal and micro-columnar forms of LuI_3_, respectively.

The discussion here emphasizes the significant impact of long-lived decay modes on decreasing the contrast in scintillator emission at synchrotron light sources. To refine the scintillator recommendations made in Table 1 ▸, the response of the recommended crystals was modelled for a range of bunch separations using the constants reported in Table 2 ▸. Four crystals (K_2_LaI_5_:Ce, Cs_2_LiLaBrCl:Ce, SrHfO_3_:Ce and BaHfO_3_:Ce) were not modelled as the relative contributions of the decay modes are not reported. Fig. 3 ▸ shows a plot of % background *versus* bunch separation for the ten modelled crystals.^5^ Here, % background is defined as the ratio of the minimum intensity observed in the scan to the maximum intensity. An increase of the background signal translates into a decreased available dynamic range. Fig. 4 ▸ shows the modelled dynamic range as a function of bunch separation.

As suggested by the experimental data in Figs. 1 ▸ and 2 ▸, the modelled curves show that the absence of dominant long-lived decay constants allows the bunch separation to be brought closer together. The calculations show that LuAG:Ce should be used with caution in time-resolved experiments on the sub-µs timescale as the dynamic range does not exceed 2 bits until the bunch separation is increased beyond 1000 ns. Dynamic experiments with moving targets using LuAG:Ce are thus expected to encounter significant difficulty in extracting quantitative contrast information (Eakins & Chapman, 2014 ▸; Jensen *et al.*, 2015 ▸). In contrast, micro-columnar LuI_3_:Ce and single-crystal LYSO:Ce deliver a dynamic range of over 16 bits (the limit of the previously identified intensified CCD cameras) at a bunch separation of 456 ns. Overlaid on Fig. 3 ▸ are dashed grey lines indicating the bunch separation in several synchrotron bunch modes suited to dynamic experiments. These suggest that to fully exploit the capabilities of the fastest time-resolved synchrotron bunch modes (*e.g.* the APS standard mode, ESRF 16BM, and PETRA III time-resolved mode) only a very small subset of crystals may be used.

The validated model may be further used to direct bunch structure development. Using the commercially available LYSO:Ce, synchrotrons could reliably employ a bunch separation of 189 ns for 1% background between bunches. At this bunch separation, experiments with LYSO would deliver a maximum dynamic range of 98 (6.6 bits). With periods of 1873 ns and 2816 ns, this ideal separation may be practically implemented as a ‘8 × 25-bunch mode’ (184 ns separation) and ‘15-bunch mode’ (188 ns separation) at DLS and ESRF, respectively. To maximize flux in a dynamic experiment without significant motion blur, the ‘8 × 25-bunch mode’ at DLS includes pulse trains each with 25 bunches and duration 50 ns. Notably, the 16-bunch mode (175 ns separation) already implemented at ESRF is close to the proposed separation and should see <2% background between bunches with LYSO:Ce. Furthermore, by modelling the scintillator response in this way the extent of image ghosting can be approximated in a growing number of dynamic radiography experiments (Luo *et al.*, 2012 ▸; Eakins & Chapman, 2014 ▸; Jensen *et al.*, 2015 ▸).

The inclusion of the offset parameter, *D*, in the modelled scintillator response suggests additional care should be taken to calibrate sources of background intensity in future experiments. In addition to the elimination of parasitic light signals *via* improved shielding, and more careful monitoring of sensor fluctuations, thermoluminescence can be further suppressed by faster X-ray shuttering, and care should be taken to understand the influence of X-ray exposure on the relative weighting of short- and long-lived decay modes.

While 14 promising crystals have been highlighted in the literature, it is clear that there is still scope for the further refinement of crystal performance *via* co-doping or alternative growth methods, or the development of entirely new crystals (Derenzo *et al.*, 2011 ▸). The enormous light yield of single-crystal LuI_3_ (van Loef *et al.*, 2008 ▸) is compromised by long decay mechanisms arising from the growth process. In contrast, LuI_3_ grown in the micro-columnar form (Marton *et al.*, 2014 ▸) is identified as the most promising crystal in this work, with a single scintillation mode of 28 ns.

Finally, the suitability of a 189 ns bunch structure separation with commercially available LYSO:Ce suggests that synchrotrons must push to tailor their bunch structures to user needs, and continue to lead the development of bunch chopping options, thereby further increasing user flexibility. Lastly, were camera quantum efficiencies significantly improved in the 300–400 nm range then many more rapidly decaying scintillator materials would become a viable choice, assuming appropriate optical relays are available.

## 8. Conclusion   

In summary, dynamic experiments at synchrotron light sources promise to address numerous outstanding problems in the materials science community. However, the scope of physics accessible to novel radiographic, diffraction and spectroscopic methods is limited by detector technology and, in particular, the choice of scintillator material. Fourteen crystals well suited to the dynamic experiments of interest here were identified, but only three (YAG:Ce, LuAG:Ce and LYSO:Ce) are available commercially.^6^ Further analysis of the performance of these crystals in experiment and *via* modelling highlights that LYSO:Ce is the best commercially available choice for time-resolved experiments on the sub-µs timescale, and was the only experimentally studied crystal to effectively resolve the DLS and ESRF bunch structures. Assuming the use of LYSO:Ce, new bunch modes achieving the best balance between flux and interframe-time were proposed for DLS and ESRF. The modeling framework developed here can be used to direct future dynamic experiments seeking to build on those reported previously (Luo *et al.*, 2012 ▸; Chen *et al.*, 2014 ▸; Eakins & Chapman, 2014 ▸) although an experiment-specific decay scan remains a necessary step.

## Figures and Tables

**Figure 2:**
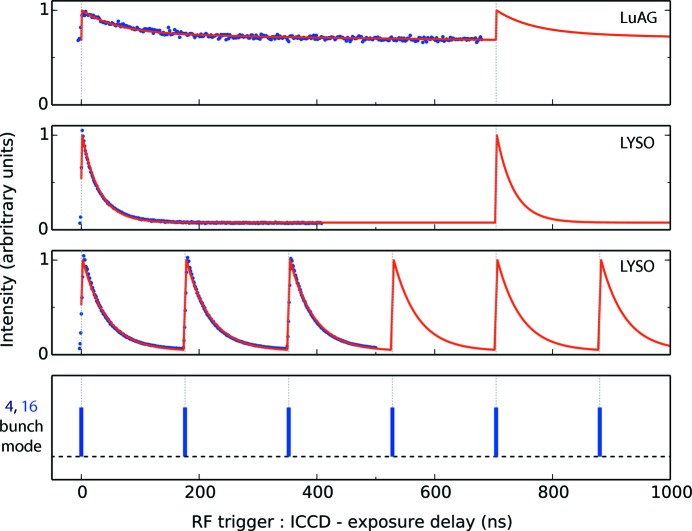
Experimental and fitted decay curves collected on beamline ID19, ESRF, with the U17-6c beam in the 4- and 16-bunch modes. Data for each scintillator are shown in a separate sub-plot. Experimental data are shown in blue. In each case the intensity range of the curve has been normalized to its maximum, collapsing the values to the range of 0–1 to aid comparison between the different materials. The modelled data, using the constants in Table 2 ▸, are shown in red. The bottom sub-plot shows an illustration of the 4- and 16-bunch modes, indicating when the X-rays were incident on the crystal. Dashed grey lines mark the position of the bunches on the experimental and modelled curves. The LuAG:Ce 4BM scan comprised 330 frames over 800 ns with a 2.81 ns exposure time. The LYSO:Ce 4BM scan comprised 330 frames over 430 ns with a 2.81 ns exposure time. The LYSO:Ce 16BM scan comprised 525 frames over 525 ns with a 5 ns exposure time, and an additional five summed on-CCD accumulations per frame to increase SNR.

**Table 1:** Scintillator materials for hard X-ray detection on the sub-µs timescale

Crystal	Density (g cm^−3^)	Emission maximum (nm)	Attenuation length (25 keV, 50 keV) (µm)	Light yield (photons MeV^−1^)	Dominant decay time (ns)	Reference
Cs_2_NaYBr_3_I_3_:Ce	4.0	425	125, 252	43000	43	Wei *et al.* (2015 ▸)
Cs_2_NaLaBr_3_I_3_:Ce	4.0	438	138, 229	58000	68	Wei *et al.* (2015 ▸)
Cs_2_LiLaBrCl:Ce	4.1	419	143, 215	50000	55	Shirwadkar *et al.* (2011 ▸)
K_2_LaI_5_:Ce	4.4	450	166, 195	52000	24	van Loef *et al.* (2003 ▸)
YAG:Ce†	4.6	550	122, 791	24000	96	Nikl (2006 ▸), Chewpraditkul *et al.* (2009 ▸)
YI_3_:Ce	4.6	532	117, 176	99000	34	van Loef *et al.* (2008 ▸)
Gd_3_Al_2_Ga_3_O_12_:Ce	4.7	550	124, 869	55000	60	Tyagi *et al.* (2015 ▸)
RdGd_2_Br_7_:5%Ce	4.7	430	77, 508	42000	45	Shah *et al.* (2002 ▸)
GdI_3_:5%Ce	5.2	552	114, 195	83000	33	Glodo *et al.* (2006 ▸), van Loef *et al.* (2008 ▸)
LuI_3_:Ce	5.6	540	91, 176	115000	33	van Loef *et al.* (2008 ▸)
Lu_3_Al_5_O_12_:Ce†	6.7	525	66, 405	27000	61	Chewpraditkul *et al.* (2009 ▸), Mares *et al.* (2012 ▸)
(LuY)Si_2_O_5_:Ce†	7.1	420	75, 461	34000	41	Pidol *et al.* (2004 ▸)
SrHfO_3_:Ce	7.6	410	45, 284	40000	42	van Loef *et al.* (2007 ▸)
BaHfO_3_:Ce	8.5	400	52, 148	40000	25	van Loef *et al.* (2007 ▸), Grezer *et al.* (2010) ▸

† Commercially available.

**Table 2:** Literature decay constants used in the scintillator response model

Scintillator	Abbreviation	*C*_1_	τ_1_ (ns)	*C*_2_	τ_2_ (ns)	*C*_3_	τ_2_ (ns)	*C*_4_	τ_4_ (ns)	*C*_5_	Reference
Cs_2_NaYBr_3_I_3_:Ce	CNYBI:Ce	0.76	43	0.09	264	0.15	1810	–	–	–	Wei *et al.* (2015 ▸)
Y_3_Al_5_O_12_:Ce	YAG:Ce	0.85	96	0.14	230	0.01	1400	–	–	–	Nikl (2006 ▸), Chewpraditkul *et al.* (2009 ▸)
YI_3_:Ce	YI_3_:Ce	0.89	34	0.11	470	–	–	–	–	–	van Loef *et al.* (2008 ▸)
Gd_3_Al_2_Ga_3_O_12_:Ce	GAGG:Ce	0.70	60	0.30	420	–	–	–	–	–	Tyagi *et al.* (2015 ▸)
RdGd_2_Br_7_:5%Ce	RGB:Ce	0.91	50	0.09	400	–	–	–	–	–	Shah *et al.* (2002 ▸)
GdI_3_:5%Ce	GdI_3_:Ce	0.33	33	0.41	91	–	–	–	–	–	Glodo *et al.* (2006 ▸), van Loef *et al.* (2008 ▸)
LuI_3_:Ce	LuI_3_:Ce	0.74	33	0.04	180	0.22	900	–	–	–	van Loef *et al.* (2008 ▸)
Lu_3_Al_5_O_12_:Ce	LuAG:Ce	0.78	61	0.12	510	0.08	2400	0.02	9900	–	Chewpraditkul *et al.* (2009 ▸), Mares *et al.* (2012 ▸)
(LuY)Si_2_O_5_:Ce	LYSO:Ce	1	41	–	–	–	–	–	–	–	Pidol *et al.* (2004 ▸)

**Table 3:** Scintillators tested on I12, DLS

Scintillator	Thickness (µm)	Supplier	Bunch mode
CRY-019	350	Crytur, CZ	686
LuAG:Ce	700	Crytur, CZ	686
LYSO:Ce	500	Crystal Photonics, FL, USA	686
YAG:Ce	80	Crytur, CZ	686

**Table 4:** Scintillators tested on ID19, ESRF

Scintillator	Thickness (µm)	Supplier	Bunch mode
LuAG:Ce	200	Crytur, CZ	4
LYSO:Ce	200	Crystal Photonics, FL, USA	4
LYSO:Ce	500	Hilger Crystals, UK	16

**Table 5:** Measured dynamic range values in the scintillator decay scans, and values of *K* and *D* obtained from the model

Scintillator	Synchrotron	Bunch mode	Energy (keV)	Maximum	Minimum	Dynamic range	*K*	*D*
CRY-019	DLS	686	55	42.0	3.11	13.5	1.621	4.18
LYSO:Ce	DLS	686	55	56.9	2.88	19.8	2.349	3.36
YAG:Ce	DLS	686	55	10.6	2.66	3.98	0.086	2.90
LuAG:Ce	ESRF	4BM	ID19 U17-6c beam	7.12	4.76	1.50	2.251	3.72
LYSO:Ce	ESRF	4BM	ID19 U17-6c beam	36.3	1.84	19.7	33.45	2.63
LYSO:Ce	ESRF	16BM	ID19 U17-6c beam	578	34.4	16.8	556.9	21.4

## Footnotes

1 Due to the short exposure times of these experiments, X-ray induced radiation damage is not significant in comparison with the damage initiated by mechanical loading methods. For example, an upper bound on X-ray heating in 1 cm^3^ aluminium is calculated, using *XOP* (del Rio & Dejus, 2011 ▸), to be less than 1 K s^−1^ in response to the white beam on beamline ID19, ESRF. This temperature rise is negligible in comparison with temperature changes of tens to hundreds of Kelvin expected during dynamic loading. X-ray probing can thus be considered a non-destructive tool for time-resolved experiments.

2 Quantum efficiency is defined as the ratio of the number of electrons produced in the photocathode for one X-ray photon incident on the photocathode.

3 A more general discussion of ideal scintillator properties, which also appeals to manufacturing concerns and higher energy experiments, is presented by Derenzo *et al.* (2003 ▸).

4 The experimental data presented in this article are available open access at http://dx.doi.org/10.5281/zenodo.48170.

5 Note that the decay response is heavily dependent on the dopant, not the crystal alone.

6 The proprietary nature of CRY-019 and it’s similar performance to LYSO:Ce means discussions have focused on the latter.

## Appendix A Measured dynamic range and fitted values of *K* and *D*

Measured maxima, minima and dynamic range values (before normalization), and fitted values of *K* and *D*, are shown in Table 5 ▸ (see §6).
